# Variable disease severity in Saudi Arabian and Sudanese families with c.3924 + 2 T > C mutation of *LAMA2*

**DOI:** 10.1186/1756-0500-4-534

**Published:** 2011-12-13

**Authors:** Claudia Di Blasi, Emanuela Bellafiore, Mustafa AM Salih, M Chiara Manzini, Steven A Moore, Mohammed Z Seidahmed, Maowia M Mukhtar, Zein A Karrar, Christopher A Walsh, Kevin P Campbell, Renato Mantegazza, Lucia Morandi, Marina Mora

**Affiliations:** 1 Division of Neuromuscular Diseases and Neuroimmunology, Fondazione IRCCS Istituto Neurologico C. Besta, Milan, Italy; 2 Division of Pediatric Neurology, Department of Pediatrics, College of Medicine, King Saud University, Riyadh, Saudi Arabia; 3 Howard Hughes Medical Institute, Division of Genetics and Manton Center for Orphan Disease Research, Children's Hospital, Boston, MA 02115, USA; 4 Department of Pathology, Roy J. and Lucille A. Carver College of Medicine, University of Iowa, Iowa City, IA 52242, USA; 5 Department of Pediatrics, Security Forces Hospital, Riyadh, Saudi Arabia; 6 Institute of Endemic Diseases, University of Khartoum, Khartoum, Sudan; 7 Department of Pediatrics and Child Health, College of Medicine, University of Khartoum, Khartoum, Sudan; 8 Howard Hughes Medical Institute and Department of Molecular Physiology and Biophysics, Roy J. and Lucille A. Carver College of Medicine, University of Iowa, Iowa City, IA 52242, USA

**Keywords:** MDC1A, LAMA2, gene, Laminin α2 chain, Merosin

## Abstract

**Background:**

Congenital muscular dystrophy type 1A is caused by mutations in the *LAMA2 *gene that encodes the laminin α2 chain, a component of the skeletal muscle extracellular matrix protein laminin-211. The clinical spectrum of the disease is more heterogeneous than previously thought, particularly in terms of motor achievement and disease progression. We investigated clinical findings and performed molecular genetic analysis in 3 families from Saudi Arabia and 1 from Sudan in whom congenital muscular dystrophy 1A was suspected based on homozygosity mapping and laminin α2 chain deficiency.

**Methods:**

We investigated 9 affected individuals from 1 Sudanese and 3 Saudi families in whom MDC1A was suggested by clinical, neuroimaging and/or pathological findings and by homozygosity mapping at the *LAMA2 *locus. Morphological and immunohistochemical analysis were performed in 3 patients from the 3 Saudi families. SSCP analysis, DNA sequencing and microsatellite analysis were carried out in the 4 index cases.

**Results:**

A previously described mutation in the *LAMA2 *gene, a homozygous T > C substitution at position +2 of the consensus donor splice site of exon 26, was found in the 4 index patients. Clinical evaluation of 9 patients from the 4 families revealed variable disease severity particularly as regards motor achievement and disease progression. Microsatellite analysis showed an identical mutation-associated haplotype in the 4 index cases indicating a founder effect of the mutation in all 4 families.

**Conclusions:**

Our data provide further evidence that the clinical spectrum of MDC1A due to a single mutation is heterogeneous, particularly in terms of motor achievement and disease progression, making it difficult to give a reliable prognosis even in patients with identical *LAMA2*-associated haplotype. The c.3924 + 2 T > C mutation to date has been found only in patients originating from the Middle East or Sudan; therefore laminin 2 chain deficiency in patients from those regions should initially prompt a search for this mutation.

## Background

Congenital muscular dystrophy type 1A (MDC1A) is an autosomal recessive neuromuscular disorder caused by mutations in the *LAMA2 *gene encoding the laminin α2 chain [1] a component of the skeletal muscle extracellular matrix protein laminin-211 [2]. Laminin-211, the most abundant laminin in muscle, is also expressed in Schwann cells, synaptic basal lamina of peripheral nerves, heart, epidermis and fetal trophoblastic tissue [3]. MDC1A is characterized by generalized hypotonia and severe muscle weakness at birth with delayed motor development, proximal joint contractures, inability to achieve independent walking, high CK levels and a clinically asymptomatic abnormality of the central white matter on brain magnetic resonance imaging (MRI) [4-6]. Several studies have also documented respiratory insufficiency, often leading to death in early childhood, and feeding difficulties. Clinical and subclinical cardiomyopathy, sensory and motor demyelinating neuropathy, and (late) external ophthalmoplegia, also occur [7]. Numerous mutations have now been identified in the *LAMA2 *gene, resulting in either complete or partial protein deficiency. However, the clinical spectrum is more heterogeneous than previously thought: a severe phenotype associated with partial expression of a laminin α2 chain isoform has been reported [8] as well as clinically mild forms with total lack of laminin α2 chain [9-11].

In the present study we report on three consanguineous Saudi Arabian families and a Sudanese family with the previously described [12] homozygous mutation c.3924 + 2 T > C in the *LAMA2 *gene. This mutation leads to aberrant splicing of exon 26 and results in an in-frame deletion of 63 amino acid residues from domain IVa of the laminin α2 chain. We also performed haplotype analysis to investigate a hypothesized founder effect of this mutation, and examined the relationship between the mutation and clinical phenotype in the 4 families.

## Methods

### Subjects

Inclusion criteria for this study were a) clinical, neuroimaging and/or pathological findings suggestive of MDC1A; and b) homozygosity at the *LAMA2 *locus if consanguinity was confirmed or suspected. We investigated 9 affected individuals from 4 families. Patients 1 and 2, a male and a female, were Sudanese; they were born to parents from the same small village and had 7 healthy siblings. Patient 3 and 4 were males born to 3 rd cousin Saudi parents; family history revealed 2 deceased affected siblings with the same diagnosis and 5 healthy siblings. Patients 5 and 6 were both males born to a 1st cousin Saudi union and had 5 unaffected brothers. Patients 7, 8 and 9 were one male and two females also born to a 1st cousin Saudi union with another 2 unaffected male and female offspring. All patients were examined by a pediatric neurologist at a tertiary referral clinic and clinical features are summarized in Additional file 1: Table S1. A muscle biopsy was performed after informed consent in patients 3, 5, 6 and 8.

Written, informed consent was obtained from the subjects or their parents/legal guardians. Research was conducted according to protocols approved by the Institutional Review Boards of Children's Hospital Boston, University of Iowa, King Saud University and Besta Neurological Institute, and in compliance with the Helsinki Declaration and local legislation.

### Immunohistochemistry

Immunohistochemical analyses were performed on muscle biopsies from patients 3, 6 and 8, from the 3 Saudi families. Patient 3's muscle was analyzed using the following anti-human laminin α2-chain monoclonal antibodies: 5H2, recognizing the G-domain (Gibco/BRL, Gaithersburg, USA), 300 kDa NCL-merosin (Novocastra, New Castle-upon-Tyne, UK), and 4H8-2 (gift to KPC from L. Sorokin) recognizing the N-terminus of the protein. Patient 8's muscle was tested only with the 300 kDa monoclonal.

In addition, the expression of dystrophin [6A9 at 1:50, Developmental Studies Hybridoma Bank (DSHB), The University of Iowa], α-dystroglycan (VIA4-1 at 1:50, Millipore), α-dystroglycan (7D11 at 1:100, DSHB), perlecan (a716 at 1:1000, Millipore) and collagen VI (5 C6 at 1:20, DSHB) were investigated in patient 3. Dystrophin, α-, β- and γ-sarcoglycan (all from Novocastra) were tested in patients 6 and 8.

Cryosections were incubated in primary antibodies diluted in PBS for 1-2 h at room temperature or overnight at 4°C. Slides were washed 5 min ×2 in PBS, then secondary antibodies were applied in PBS for 30 min-1 h at room temperature. After washing again 5 min ×2 in PBS, coverslips were mounted using ProLongGold with DAPI (Molecular Probes Inc, Eugene, OR, USA). Secondary antibodies were either goat-anti-mouse tagged with AlexaFluor488 or goat-anti-rat tagged with AlexaFluor594 (both from Molecular Probes).

### Molecular analyses

Genomic DNA was extracted from peripheral blood and analyzed by the polymerase chain reaction touchdown method using oligonucleotide primers flanking the intron-exon junctions of all 65 *LAMA2 *exons (exon numbering according to the Leiden muscular dystrophy database) [10]. Aberrant conformers, identified by single-strand conformation polymorphism (SSCP) analysis, were sequenced using an ABI Prism 3100 analyzer (Applied Biosystems, Foster City, CA, USA).

Total RNA was prepared from skeletal muscle using TRI Reagent (Ambion, Inc. Austin, TX). The isolated RNA was reverse transcribed using a First-Strand cDNA Synthesis Kit (Roche Molecular Biochemicals, Basel, Switzerland), the resulting cDNA was amplified by reverse transcriptase PCR (RT-PCR) and sequenced using appropriate primers.

### Haplotyping

Haplotype analysis was performed on genomic DNA using microsatellite markers provided by Genethon human genetic linkage map [13] and flanking the LAMA2 gene: upstream (D6S407) and downstream (D6S1620, and D6S1705); and six intragenic polymorphisms: G1905A, A2848G, G5515A, A5551G, C5579A and G6286A [14]. Allele frequency of polymorphisms in a reference population are reported in Guicheney et al. [14].

## Results

Clinical findings are summarized in Additional file 1: Table S1. All patients were floppy babies and manifested with generalized hypotonia. Severe proximal weakness was also present from birth or developed within the first 6 months. In all cases motor milestones were delayed. Five of the 9 patients achieved independent walking at 3, 3.5 and 4 years, which was retained at least until 8, 11, 12 or 13 years respectively, when last seen. Patients 3-6, who never walked, could sit or stand with or without support. CK was normal in patient 9, and slightly to markedly increased in all others.

**Figure 1:**
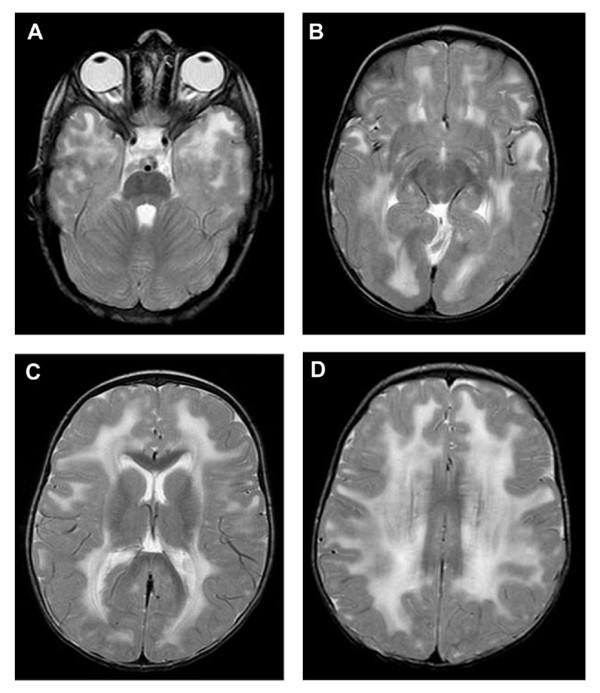
**T2-weighted MRI images of patient 7, taken at the age of 19 months**. Showing abnormal periventricular and sub-cortical white matter signal.

Electrocardiogram (performed in 5 patients) and echocardiogram (performed in 4 patients) were normal; cardiac signs were reported in none of the patients. Respiratory support was necessary in patient 3 who died of respiratory failure at 7 years and in patient 5 who also died of respiratory failure at 16 years. No mental retardation, epilepsy or eye abnormalities were observed. MRI or CT, performed in 8 patients, revealed white matter changes in all cases (Figure 1). Patients 3 and 6 underwent EMG, and myopathic features were found in both cases.

**Figure 2:**
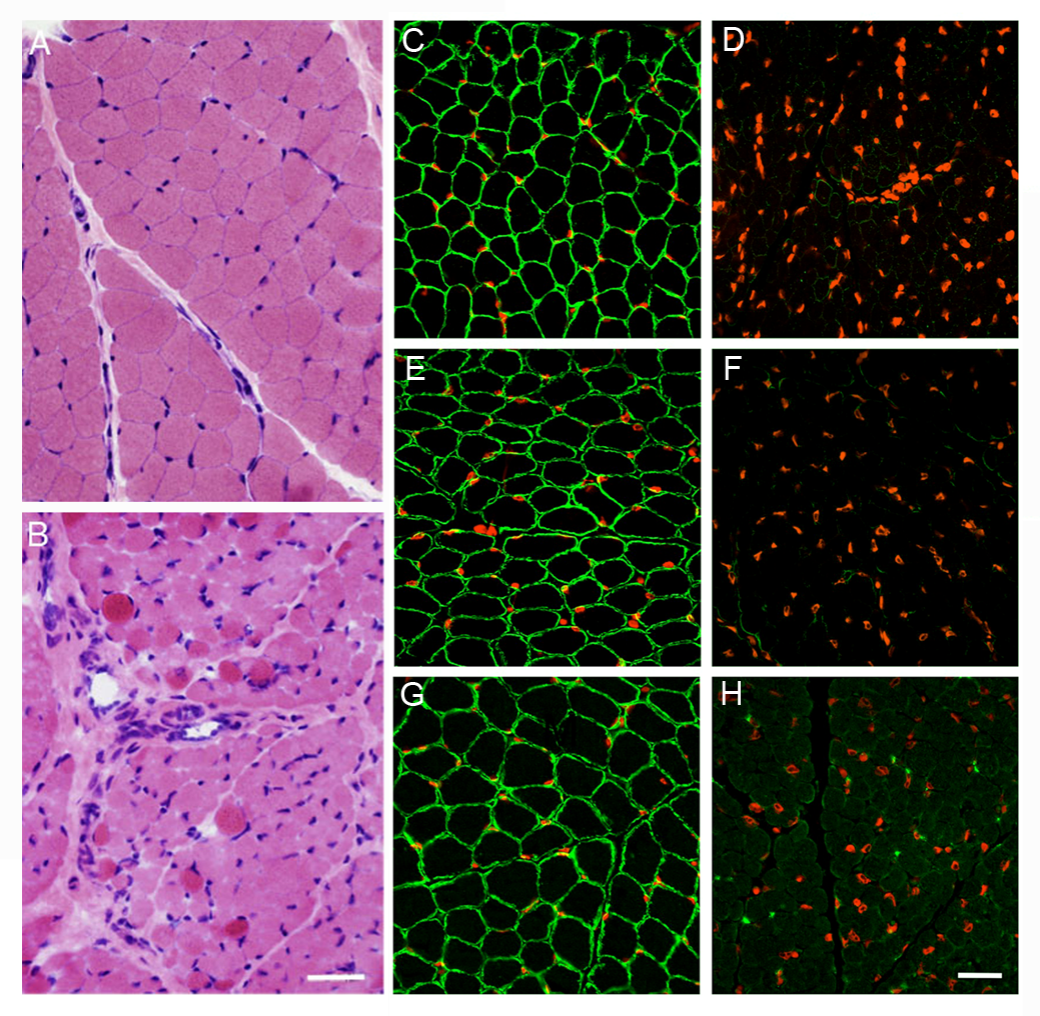
**H&E in control (**A**) and MDC1A (**B**) muscle (bar = 25 μm); and immunohistochemistry (**C**-**H**) in control (**C**,**E**,**G**) and patient 3 muscle (**D**,**F**,**H**) laminin α2 chain with 5H2 (**C**,**D**), 300 kDa (**E**,**F**) and 4H8 (**G**,**H**) antibodies**. Nuclei are stained with DAPI, pseudo-colored in red (bar = 50 μm).

A muscle biopsy from patient 3 (taken at 5 weeks) showed mild dystrophic features; whereas muscle biopsies from patients 5 (taken at 3 years and 2 months), 6 (taken at 5 years) and 8 (taken at 4 years) showed marked dystrophic features. Immunofluorescence analysis of the laminin α2 chain was only performed in patient 3 (with 3 antibodies) and in patient 8 (1 antibody); this revealed greatly reduced staining intensity with the 3 antibodies in patient 3 (Figure 2) and with the single antibody in patient 8 (data not shown). Dystrophin and dystrophin-associated proteins were normal in patients 3, 6 and 8.

SSCP analysis in patients 1, 4, 5 and 7 (index cases) revealed abnormal conformers in exon 26. The exon was sequenced and showed a T > C substitution at position +2 of the consensus donor splice site. This mutation has been described previously in two siblings from a consanguineous Saudi family unrelated to ours [12]. Direct sequencing of the cDNA revealed a 189 bp in-frame deletion, corresponding to aberrant skipping of the whole of exon 26, with loss of 63 amino acids (residues 1246-1308) from domain IVa of the protein (See Additional file 2: Figure S1).

Because of the consanguinity in the Saudi families and presumed consanguinity in the Sudanese family, it is likely that all affected siblings are homozygous for this mutation.

Investigation of flanking and intragenic microsatellite markers of the *LAMA2 *gene in the 4 index cases indicated an identical mutation-associated haplotype (containing the mutation) between marker D6S407 and intragenic polymorphism G6286A (data not shown), suggesting remote consanguinity and a founder effect in all 4 families. By contrast, the downstream markers (D6S1620, and D6S1705) were not identical in the 4 cases.

## Discussion

In 3 families from Saudi Arabia and 1 from Sudan we have identified a previously reported homozygous c.3924 + 2 T > C mutation in the *LAMA2 *gene associated with variable clinical phenotype. The main phenotypic differences among our 9 cases regard motor achievement. Patients 1 and 2 from the Sudanese family walked at 4 years; patients 7, 8 and 9 from a Saudi family walked at 3, 3.5 and 4 years, respectively. These 5 patients were still walking at 8, 11, 12 and 13 years when last seen, while patients 3, 4, 5, and 6 from the 2 other Saudi Arabian families never walked.

CK levels were also variable, being very high (5.5 - 16.8 × normal) in patients 3, 6, 7, and 8; high (1.2 - 4.4 × normal) in patients 1, 2, 4 and 5; and normal in patient 9.

Features common to all were floppiness in infancy, delayed motor milestones or failure to achieve walking, brain white matter attenuation on MRI or CT (not done in 1 patient) and development of joint contractures/foot deformities. Eye and cardiac abnormalities were not observed. In patients 3 and 5 respiratory compromise was present, and both died of respiratory failure at 7 and 16 years, respectively.

In 1997 Allamand et al. [12] reported on a brother and sister from a consanguineous Saudi family with the same *LAMA2 *mutation as found in our patients (but characterized as 3973 +2 T > C according to the previous *LAMA2 *nucleotide sequence numbering). Both were mildly affected: the boy at 3.5 years had muscle hypotonia and inadequate head control, but walked at 26 months; his younger sister also had hypotonia from early infancy and poor head control and achieved walking at 3 years 8 months. Both had slightly reduced laminin α2 chain expression as investigated by 2 antibodies recognizing the G domain, and highly reduced expression using an antibody recognizing the N terminal. These results demonstrated for the first time that use of more than one antibody can provide valuable indications as to what domain(s) of the laminin α2 chain may be affected in CMD patients. Thereafter, this procedure became the standard method for staining muscle of patients with CMD and has also been used for prenatal diagnosis.

By contrast, in our patient 3, analyzed with 3 different antibodies, laminin α2 chain expression was markedly reduced: clearly more so than the cases described in Allamand et al. [12]. He was the most severely affected of our cases.

It is noteworthy that our cases show a much wider clinical spectrum than suggested by the siblings described by Allamand et al. [12]: from the very severe patient 3 and his brother (patient 4 who never walked or sat unaided), to patients 1, 2, 8 and 9 who were still walking at latest examination.

We previously reported two siblings from a consanguineous family with partial laminin α2 chain deficiency due to an in-frame deletion and exceptionally mild clinical manifestations [15]. The proband was a 39 year-old man whose symptoms (difficulty in running and jumping), first appeared at age 15 years and worsened very slowly. When examined, the proband's sister was found to have a similar though even milder clinical picture. Siala et al. [16] reported that clinical severity differed between two siblings with the same out-of-frame mutation in the *LAMA2 *gene, which was unrelated to laminin α2 chain expression (completely undetectable in both cases). Such clinical variability implies the presence of other genetic or epigenetic factors able to influence disease phenotype (see Heydemann et al. [17]).

The data of the present study provide further evidence that the clinical spectrum of MDC1A is more heterogeneous than previously thought; motor achievement and disease progression are particularly variable, making it difficult to formulate prognoses even in patients with an identical *LAMA2*- associated haplotype. Modifier genes, such as those coding for proteins that interact with laminin α2, and/or epigenetic factors, for instance those involved in regulatory signaling functions, are likely to contribute to the observed phenotypic variability. A drawback of the present study is that muscle biopsies were stained for laminin α2 in only 2 cases and extensive immunohistological characterization was only possible in one case.

We emphasize, finally, that the identical intragenic polymorphisms and upstream microsatellite markers of the *LAMA2 *gene in our patients, strongly suggest a founder effect. The mutation probably originated in Saudi Arabia since studies on severe childhood autosomal recessive muscular dystrophy (SCARMD), the common form of muscular dystrophy in North Africa and the Arabian Peninsula [18-21] indicate that affected families from the same tribe migrated from central Saudi Arabia to the Sudan - crossing the Red Sea - in the 12th and 13th centuries [22]. Furthermore, in Saudi Arabia the mutation may not be rare since we have recently learned (personal communication of Dr. Thomas L. Winder to SAM) of 3 Saudi patients with this mutation, 2 of whom are homozygous and one who is heterozygous. A few more cases, all originating from the Middle East, are reported in the Leiden database http://www.dmd.nl/. The clinical implication is that a laminin α2 chain deficiency in Middle Eastern or Sudanese patients should initially prompt a search for the c.3924 + 2 T > C mutation in the *LAMA2 *gene.

## Conclusions

Our data provide further evidence that the clinical spectrum of MDC1A due to a single mutation is heterogeneous, particularly in terms of motor achievement and disease progression, making it difficult to give a reliable prognosis even in patients with identical *LAMA2*-associated haplotype.

The c.3924 + 2 T > C mutation to date has been found only in patients originating from the Middle East or Sudan; therefore laminin 2 chain deficiency in patients from those regions should initially prompt a search for this mutation.

## Competing interests

The authors declare that they have no competing interests.

## Authors' contributions

CDB carried out the molecular and microsatellite analysis and drafted the manuscript; EB performed molecular analysis; MAMS evaluated clinical and neurologic features of the patients, collected DNA and muscle biopsies and revised the manuscript critically for important intellectual content; MCM carried out homozygosity mapping and linkage analysis; SAM performed immunochemical evaluation of muscle biopsies and prepared the figures; MZS, MMM and ZAK evaluated neurologic, neurophysiologic and MRI features; CAW and KPC supervised the study and critically revised the manuscript; LM and RM contributed to writing the clinical reports, revised the manuscript critically and provided financial support; MM was responsible for study design, supervised the study and manuscript completion. All authors read and approved the final manuscript.

## Supplementary Material

Additional file 1 **Table S1**. Clinical findings. Click here for file

Additional file 2 **Figure S1**. Sequencing of genomic DNA from control (**A**) and patient (**B**) showing the T > C transition at position +2 of the consensus donor splice site of exon 26. Direct sequencing of the cDNA revealing a 189 bp in-frame deletion, corresponding to aberrant skipping of the whole of exon 26: (**C**) control and (**D**) patient. Click here for file
