# Supplementary material for: Variable disease severity in Saudi Arabian and Sudanese families with c.3924 + 2 T > C mutation of LAMA2
Source: BMC Res Notes. 2011 Dec 13;4:534. doi: 10.1186/1756-0500-4-534 (PMC3278494; doi:10.1186/1756-0500-4-534)
Supplement: Additional file 1 — Table S1. Clinical findings. [file 1756-0500-4-534-S1.DOC]

**Table 1. Clinical findings**

| **Patient / Sex** | **Age at onset** | **Age walked** | **Maximum motor ability** | **Age first examination** | **Muscle hypertrophy** | **Joint contractures deformities** | **Age last seen** | | **CK^1^ (age when done)** | **Brain MRI/CT** | **Laminin α2 chain** | **Other** |
| --- | --- | --- | --- | --- | --- | --- | --- | --- | --- | --- | --- | --- |
| 1/M (family 1) | Birth | 4 y | Still walking at 13 y | 11 y | Calf muscles | None | 11 y | | ^2^603 (11 y) | White matter attenuation (MRI) | Not done | Nothing remarkable |
| 2/F  (family 1) | Birth | 4 y | Still walking at 11 y | 9 y | Calf muscles | Mild scoliosis concave to the right | 9 y | ^2^349 (9 y) | | White matter attenuation (MRI) | Not done | Nothing remarkable |
| 3/M (family 2) | Birth | Never | Sits with support | Birth | No | No | 5 y; died at 7 y | ^2^3270 (19 d); 1990 (1 m) | | White matter attenuation (MRI) | Reduced (3 abs) | EEG normal |
| 4/M (family 2) | 2 m | Never | Cannot walk or sit by himself | 2 m | No | Hips, knees and ankles with bilateral equinovarus | 6 y, 9 m | ^2^864 (8 m) | | Not done | Not done | Nothing remarkable |
| 5/M (family 3) | Birth | Never | Stands with support | 3 y, 2 m | Calf and hamstrings | Hips, knees, and elbows with equinovarus, kyphoscoliosis | 16 y; died at16 y | ^2^280 (3 y, 2 m) | | White matter hypoattenuation (CT). White matter attenuation (MRI) | Not done | ^3^ECG: normal at 4 y, BAER: upper brainstem bilateral involv: R>L, electroretinogram: normal, VEP normal |
| 6/M (family 3) | Birth | Never | Sits without support | 13 m | No | Hips, knees, elbows and heel cords | 18 y | ^2^1063 (5 y) | | White matter attenuation (MRI) | Not done | ECG normal at 5 y |
| 7/M  (family 4) | < 6 m | 3 y | Walked till 13 y | 7 y | Calf muscles | Bilateral equinovarus | 13 y | ^2^2506 (7 y) | | Diffuse white matter attenuation (MRI: 6 y) | Not done | ECG normal at 6 y |
| 8/F (family 4) | < 6 m | 3.5 y | Still walking | 4 y | Calf muscles | Right equinovarus | 12 y | ^22^1929 (4 y) | | Accentuated white matter hypodensity (CT: 3 y) | Reduced (1 ab) | ECG normal at 3 y |
| 9/F (family 4) | < 6 m | 4 y | Still walking | 19 m | Calf muscles | None | 8 y | ^2^49 (19 m) | | Diffuse white matter attenuation (MRI:19 m) | Not done | ECG normal at 2 y |

^1^U/L; ^2^Normal values vary with laboratory from <110 to ≤232; ^3^Abbreviations: BAER, brain auditory evoked responses; ECG, electrocardiogram; ERG, electroretinogram; L, left; R, right; VEP, visual evoked potentials.
