# Supplementary material for: Variable disease severity in Saudi Arabian and Sudanese families with c.3924 + 2 T > C mutation of LAMA2
Source: BMC Res Notes. 2011 Dec 13;4:534. doi: 10.1186/1756-0500-4-534 (PMC3278494; doi:10.1186/1756-0500-4-534)

**Supplementary Figure 1**

Sequencing of genomic DNA from control (A) and patient (B) showing the T>C transition at position +2 of the consensus donor splice site of exon 26.

Direct sequencing of the cDNA revealing a 189 bp in-frame deletion, corresponding to aberrant skipping of the whole of exon 26: (C) control and (D) patient.

# Exon 26

A

GtaagcacaagaactttaatgtcaagtgagaacaagataaaatctttttagaatcacaccatttggagatttatccaattcctcattcttctttttattttgtcagTTGATGGCCTATGGGGGCAAACTCAAGTATGCAATCTATTTCGAGGCTCGGGAAGAAACAGGTTTCTCTACATATAATCCTCAAGTGATCATTCGAGGTGGGACACCTACTCATGCTAGAATTATCGTCAGGCATATGGCTGCTCCTCTGATTGGCCAATTGACAAGGCATGAAATTGAAATGACAGAGgtaaagttagtcattgtttggtgcaaagataccaatcaatggttttgcattcagttttgtcatagtgatttctcttcttgttaacag

B

GtaagcacaagaactttaatgtcaagtgagaacaagataaaatctttttagaatcacaccatttggagatttatccaattcctcattcttctttttattttgtcagTTGATGGCCTATGGGGGCAAACTCAAGTATGCAATCTATTTCGAGGCTCGGGAAGAAACAGGTTTCTCTACATATAATCCTCAAGTGATCATTCGAGGTGGGACACCTACTCATGCTAGAATTATCGTCAGGCATATGGCTGCTCCTCTGATTGGCCAATTGACAAGGCATGAAATTGAAATGACAGAGgcaaagttagtcattgtttggtgcaaagataccaatcaatggttttgcattcagttttgtcatagtgatttctcttcttgttaacag

C


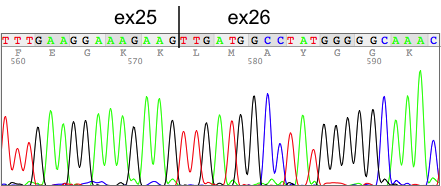


D


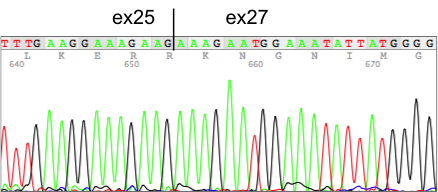

Supplement: Additional file 2 — Figure S1. Sequencing of genomic DNA from control (A) and patient (B) showing the T > C transition at position +2 of the consensus donor splice site of exon 26. Direct sequencing of the cDNA revealing a 189 bp in-frame deletion, corresponding to aberrant skipping of the whole of exon 26: (C) control and (D) patient. [file 1756-0500-4-534-S2.DOC]
